# A fine-tuned defense at the pea root caps: Involvement of border cells and arabinogalactan proteins against soilborne diseases

**DOI:** 10.3389/fpls.2023.1132132

**Published:** 2023-02-09

**Authors:** Mélanie Fortier, Vincent Lemaitre, Alexia Gaudry, Barbara Pawlak, Azeddine Driouich, Marie-Laure Follet-Gueye, Maïté Vicré

**Affiliations:** Univ Rouen Normandie, GLYCOMEV UR 4358, SFR Normandie Végétal FED 4277, F-76000, Rouen, France

**Keywords:** associated cap-derived cells (AC-DCs), *Aphanomyces euteiches*, arabinogalactan-proteins (AGPs), root border cells, *Pisum sativum, L.*, root defense, root extracellular trap (RET), root disease

## Abstract

Plants have to cope with a myriad of soilborne pathogens that affect crop production and food security. The complex interactions between the root system and microorganisms are determinant for the whole plant health. However, the knowledge regarding root defense responses is limited as compared to the aerial parts of the plant. Immune responses in roots appear to be tissue-specific suggesting a compartmentalization of defense mechanisms in these organs. The root cap releases cells termed root “associated cap-derived cells” (AC-DCs) or “border cells” embedded in a thick mucilage layer forming the root extracellular trap (RET) dedicated to root protection against soilborne pathogens. Pea (*Pisum sativum*) is the plant model used to characterize the composition of the RET and to unravel its function in root defense. The objective of this paper is to review modes of action of the RET from pea against diverse pathogens with a special focus on root rot disease caused by *Aphanomyces euteiches*, one of the most widely occurring and large-scale pea crop diseases. The RET, at the interface between the soil and the root, is enriched in antimicrobial compounds including defense-related proteins, secondary metabolites, and glycan-containing molecules. More especially arabinogalactan proteins (AGPs), a family of plant extracellular proteoglycans belonging to the hydroxyproline-rich glycoproteins were found to be particularly present in pea border cells and mucilage. Herein, we discuss the role of RET and AGPs in the interaction between roots and microorganisms and future potential developments for pea crop protection.

## 1 Introduction

Legume seeds are an important source of dietary protein, carbohydrates, minerals, vitamins, and antioxidants presenting many advantages and great potential for human and animal nutrition. Garden pea (*Pisum sativum* L.) is one of the most widespread food legume crops cultivated in more than 90 countries all over the world (FAO, 2018) for its nutritional value and high-quality vegetable proteins. Its consumption is recognized to improve human diet and health by reducing cholesterol or preventing stomach cancer (Nazir et al., 2022). Several studies were dedicated to unravel pea proteins composition and properties making pea a widely used source of commercial proteins attracting attention in food industry (Karaca et al., 2011; Sun and Arntfield, 2012; Burger and Zhang, 2019). As compared to soybean (*Glycine max*) proteins, pea proteins present the advantage for food products to be deprived of allergen and being without genetic modification (Day, 2013; Krefting, 2017). Furthermore, pea is also a culture of interest as it does not require nitrogen fertilizer for its growth due to its capacity to fix atmospheric nitrogen *via* symbiosis with rhizobia thereby enriching the soil in nitrogen (Foyer et al., 2016). Therefore, pea is considered as an economical and environmental friendly crop, which improves crop productivity by reducing the demand for external nitrogen fertilizers in many farming systems. Despite its high nutritional value and remarkable advantages, the yield of the pea crop gets drastically reduced due to root diseases. More especially, *Aphanomyces euteiches* responsible of the root rot disease causes devastating damages to pea crops and significant economic losses (Gaulin et al., 2007). *A.euteiches* is particularly destructive on spring pea crops but also on other legumes such as green bean (*Phaseolus vulgaris*) or lentil (*Lens culinaris*). There is currently no effective way to control *A. euteiches* and root rot spreading, as neither the phyto-chemicals nor the resistant varieties are available. Avoidance of infested fields based on crop rotation remains the main used method to limit the spread of this disease. However, the long-term survival of *A. euteiches* oospores in the soil up to ten years is a serious limitation of this cropping management practice (Gibert, 2021). This results on an increasing need for new cropping systems and/or cultivar selection for pea producers in order to maintain sufficient yields. To this end, it is necessary to unravel the molecular dialogue at the root tip between pea and pathogens. This review summarizes current knowledge about the role of the root extracellular trap (RET) in pea root protection and presents the more promising strategies to control root disease with a special focus on root rot disease caused by *A. euteiches*.

## 2 Pea: the plant model to decipher the role of border cells in root defense

**Figure 1:**
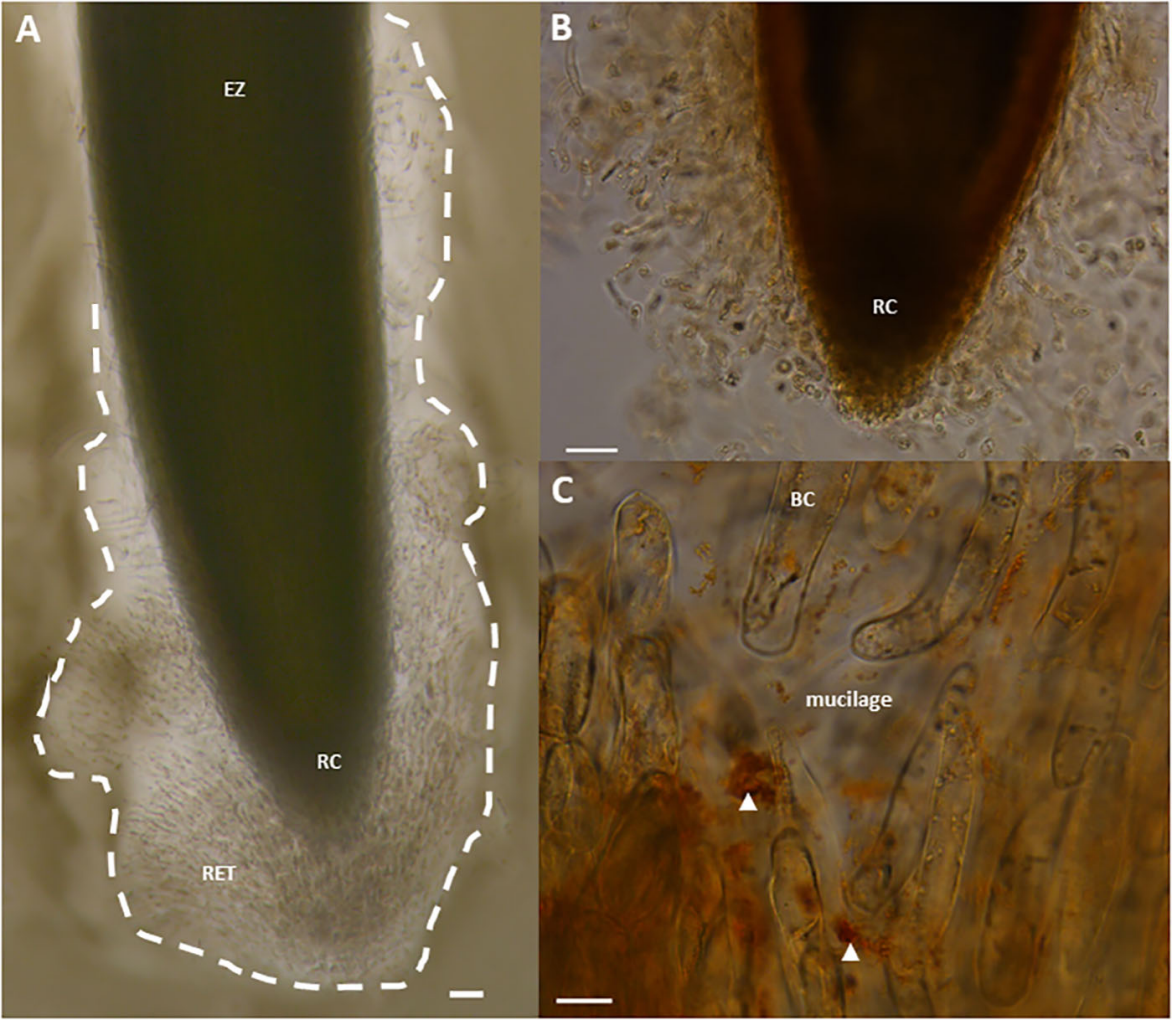
Light micrographs showing border cells and mucilage released by pea (*Pisum sativum* var. Astronaute) root tips, forming the RET, stained with India ink **(A)**, or with the β-glucosyl-Yariv reagent **(B, C)**. Note the observation of brown/red aggregates, indicated by white arrowheads, and signaling the presence of AGPs **(C)**. BC, border cell; EZ, elongation zone; RC, root cap; RET, Root Extracellular Trap. Scale bars, 100 µm **(A, B)** and 20 μm **(C)**.

**Figure 2:**
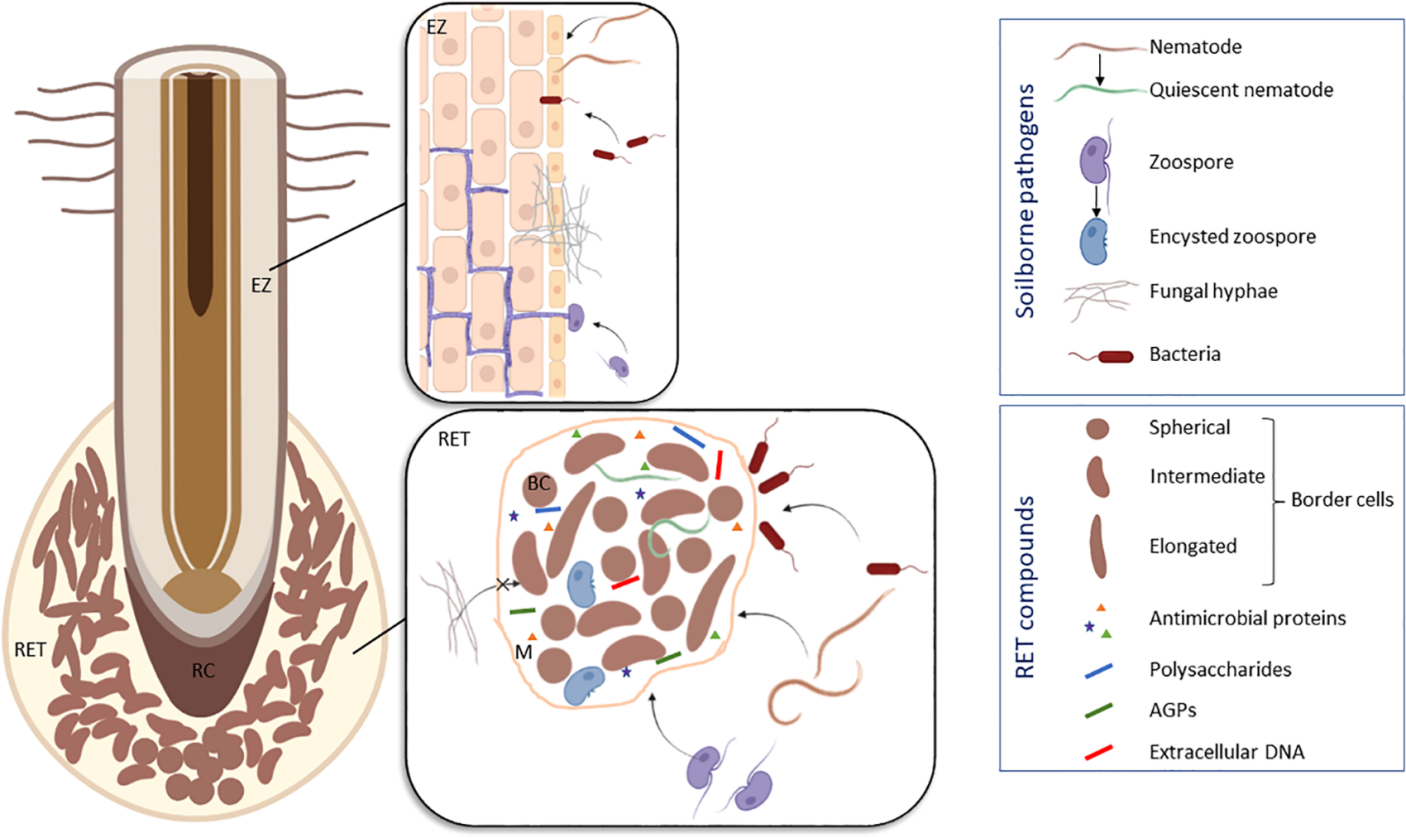
Schematic model illustrating the different modes of interaction between pea (*Pisum sativum*) roots and soilborne pathogens based on the results of Hawes et al., 2000; Gunawardena and Hawes, 2002; Gunawardena et al., 2005; Cannesan et al., 2011 and Zhao et al., 2000. To protect the root tip, the RET compounds are able to attract nematodes (e.g. *Meloidogyne incognita*) and to induce their quiescence, to trap oomycetes (e.g. *Aphanomyces euteiches*) and to induce their encystment, to prevent penetration of fungi (e.g. *Nectria haematoccoca*) and to exclude bacteria (e.g. *Pseudomonas aureofaciens*). Infection sites are usually located in the elongation zone of the root. BC, border cell; EZ, elongation zone; M, mucilage; RC, root cap; RET, Root Extracellular Trap. Figure created in BioRender.com.

Plant defenses were mainly studied on the foliar parts whereas the belowground system remained ignored due to the difficulty of its access and the complexity of root-microbe interactions involving a diversity of beneficial and harmful soilborne microorganisms (Erb et al., 2011; Balmer et al., 2013). This is particularly true and crucial for legume roots which need to distinguish between mutualistic microbes and pathogens in order to allow symbiotic microorganisms such as rhizobia to colonize root tissues forming root nodules (Bozsoki et al., 2017). Immune signaling and responses in roots are not only different from leaves but are also compartmentalized within the different zones of this organ (Chuberre et al., 2018). Root elongation zone is recognized as the main entrance area for most of soilborne pathogens whereas root tip rarely develops lesions at early stages of infection (Gunawardena et al., 2005). This protection is due to atypical cells termed root “associated cap-derived cells” (AC-DCs) released from the root cap. “AD-DCs” are essential in root defense and comprise different cell populations according to their mode of detachment from the root: “root border cells” are AC-DCs released individually as in pea (**Figure 1**), whereas “border-like cells” are AC-DCs forming layers of cells that remain attached to the root cap as in *Arabidopsis thaliana* (Hawes, 1990; Vicré et al., 2005). The production of border cells was first described in pea (Hawes et al., 1998). Border cells, originally called “sloughed root cap” were defined as “living cells programmed to separate individually from the periphery of roots into the external environment” (Hawes and Pueppke, 1986). Border cells remain in close vicinity of the root cap as they are embedded in a thick mucilage acting as a “glue”. Upon contact with water, the mucilage-that can hold 1,000 times its weight in water-swells leading to dispersion and release of border cells into the rhizosphere (Hawes et al., 1998). Experimentally, border cells can be easily visualized under binoculars by placing the root tip into water; the cells become dispersed in response to gentle agitation (Hawes and Lin, 1990). As they separate from pea root cap, border cells differentiation from root cap peripheral cells into border cells is accompanied by a switch in gene expression leading to the synthesis of a set of proteins and metabolites involved in root defense (Brigham et al., 1995; Wen et al., 2007; Wen et al., 2009). An array of 100 extracellular proteins was found to be released while border cell separation proceeds (Brigham et al., 1995). At the frontier between root and soil, root border cells are key elements controlling root interactions with microorganisms (**Figure 2**). Their functions are diverse according to both plant species and microorganisms. In pea, root border cells were clearly shown to be involved in root tip protection against *Nectria haematoccoca* infection (Gunawardena and Hawes, 2002; Gunawardena et al., 2005). Despite a formation of a mantle of hyphae covering the surface of the root tip, border cells detached from the root together with the pathogens leaving the root cap deprived of mycelium. This mostly happens at early stages of infection. Extracellular proteins secreted by border cells such as β-1-3,3 proteins as well as extracellular DNA were shown to contribute to pea root protection against *N. haematoccoca*. Border cells from pea were also shown to act as a “lure” against some species of fungi and nematodes by specifically attracting pathogens to the root tip for better neutralization (Hawes et al., 2000). When inoculating pea root with the pathogenic nematode *Meloidogyne incognita*, second-stage juveniles (J2) accumulated specifically at the root tip unsheathed by border cells. After a few minutes of contact with pea border cells, J2 lost their motility and entered into reversible quiescence (Zhao et al., 2000). Whereas J2 rapidly accumulated within clumps of *in vitro* detached border cells, no attraction was observed using pea root exudates. Reversible quiescence induced by pea root border cells was also reported with other nematodes but it should be noted the levels varied according to the green pea cultivars tested (Hiltpold et al., 2015). Such positive chemotaxis of nematodes by root border cells was species-dependent for the legumes studied: no attraction was found to occur in snap bean whereas repulsion was induced by alfalfa (Zhao et al., 2000). It is therefore of high interest to identify the nature of molecules produced and secreted by pea root border cells involved in chemotaxis and able induce a state of reversible quiescence of parasitic nematodes. Root border cells produce a quite abundant extra-cellular mucilage that forms a protective shield at the root tip. Such halo of mucilage was even more induced upon inoculation of wheat border cells with *Agrobacterium tumefasciens*. In this case, the mucilage allows exclusion of bacteria from the surface of border cells. However, such mechanisms have not been reported in pea border cells and *A. tumefasciens* were able to access to the border cells surface. Pea root infection by *A. euteiches* occurs mainly in the elongation and root hair areas with the exception of the root cap and border cells. In contrary to what was reported regarding infection with *N. haeamatococca*, border cells surface was not covered by the presence of mycelium and encysted zoospores (Cannesan et al., 2011). Such findings are in support of the hypothesis that root border cells in pea are involved in local defense of the root tip against *A. euteiches* preventing root cap colonization at early stages of infection. More specifically, we speculated that spherical border cells are the more active cells involved in root defense as compared to intermediate and elongated border cells. Defense mechanisms provided at the root tip by border cells appeared particularly complex as different border cells populations from pea might not be involved at the same level in root protection.

Border cells from pea present selective interactions with soilborne microorganisms by attracting, repelling or even inhibiting the growth of fungal, bacterial or oomycete pathogens (**Figure 2**) (Sherwood, 1987; Hawes and Brigham, 1992; Wen et al., 2007; Wen et al., 2009; Cannesan et al., 2011; Cannesan et al., 2012). Zhu et al. (1997) demonstrated that the ability of pea border cells to induce *in vitro* expression of bacterial gene required for the establishment of plant-microbe associations was selective. Little to no *vir* (*A. tumefasciens*) gene or *pkz* (pathogenic *Pseudomonas aureofaciens*) gene induction occurred in response to co-cultivation of these pathogenic bacteria with border cells of pea. However, the presence of pea border cells induced a significant increase in the expression of nod genes of *Rhizobium leguminosarum bv viciae*, a strain that nodulated pea (Zhu et al., 1997). It is thus remarkable that border cells from pea can influence expression of some genes from symbiotic bacteria but not others. It was then proposed that border cells are important actors in controlling the ecology of the rhizosphere by regulating growth and gene expression in microbial populations (Hawes, 1990; Hawes and Brigham, 1992). It also became obvious that root border cells do not act alone but in synergy with the surrounding mucilage layer to provide root protection against pathogens. Based on the Neutrophil Extracellular Trap (NET) described in mammals, the Root Extracellular Trap (or RET) model was proposed to explain the interconnection between AC-DCs and the mucilage (Driouich et al., 2013). The mucilage is a fibrillary structure forming a web that enhances the adhesion of microorganisms and facilitate pathogen neutralization by defense molecules produced and released by AC-DCs. We have postulated that fine-tuned communications are connecting AC-DCs throughout the RET in a similar way to the biofilms formed by bacteria (Driouich et al., 2019). The molecular events involved in the structuration and cell communication at the RET level remain to be in-depth established in order to unravel belowground defense mechanisms of the pea root tip.

## 3 Molecular dialogue at the pea root tip: a focus on glycomolecules

It has been estimated that approximately 20 to 25% of the total reduced carbon released by maize roots is in the form of high molecular weight root mucilage (Chaboud, 1983). Root mucilage exocytosis from border cells of different plant species such as maize or pea mainly consist mainly of polysaccharides including hemicellulosic compounds and pectins (Chaboud, 1983; Rougier and Chaboud, 1985; Vicré et al., 2005; Mravec et al., 2017). Homogalacturonans are essential components of pea root mucilage and are involved in cementing root border cells together. It has been reported that partial inhibition of the pectin methylesterase (*rcpme1*) in transgenic pea roots was correlated to the formation of a cohesive clump of border cells that could not separate from the root cap (Wen et al., 1999; Durand et al., 2009; Mravec et al., 2017). Correct expression of *rcpme1* in the pea root caps is thus necessary to provide border cells separation and release from the root cap showing the importance of the degree and pattern of methyl esterification of homogalacturonan in these events. The presence of xylogalacturonan (XGA) epitope recognized by the mAb LM8 was also associated with pea border cells detachment and was found to be released within extracellular bodies at the root surface in the mucilage (Mravec et al., 2017). Although the precise role of XGA remains to be clearly established, the presence of xylose residues prevents polysaccharides to be enzymatically degraded by pathogenic agents upon root infection (Jensen et al., 2008). Consequently, XGA could contribute to the mechanical barrier preventing microbial invasion at the root tip. Interestingly, Knee et al. (2001) reported that monosaccharide composition from pea root mucilage appeared to contain specifically high amount of arabinose (Ara) and galactose (Gal) possibly related to the presence of arabinogalactan proteins (AGPs). Cannesan et al. (2012) detected the presence of epitopes associated with AGPs at the border cell surface and within the mucilage. The monosaccharide composition and profiles of AGPs from the pea root cap, border cells and mucilage were distinct from the rest of the root system and were found to be species-specific. Furthermore, experimental data were consistent with the hypothesis that AGPs from pea root tips interfere with *in vitro* cell cycle of *A. euteiches*. *In vitro* assays showed that AGPs isolated from pea root cap and border cells were able to attract zoospores and inhibit subsequent cyst germination. AGPs are thought to be essential elements in root-microbe interactions in both pathogenic and beneficial microorganisms (Xie et al., 2012; Nguema-Ona et al., 2013). Xie et al. (2012) demonstrated the function of AGPs from pea root in controlling *in vitro* surface attachment of *Rhizobium leguminosarum*. The authors suggest that AGPs could bind to one or both bacteria poles, thereby promoting their polar attachment to the root surface. A role of AGPs in *Agrobacterium* and *Rhizobium* adhesion to the root was previously reported in *Arabidopsis thaliana* supporting the importance of these proteoglycans in microorganisms attachment but the mechanisms of actions remains to be clarified (Gaspar et al., 2004; Vicré et al., 2005). However, it cannot be excluded that a complex including AGPs and different components could be involved in bacterial root adhesion. Interactions between AGPs and pectins such as homogalacturonans have been previously shown to occur although the exact linkage type are not determined (Oosterveld et al., 2002; Immerzeel et al., 2006; Cannesan et al., 2012). Classical AGPs bind reversibly to Ca^2+^ in a pH-dependent manner by glucuronic carboxyl groups. Ca^2+^-driven cross-linking between the carboxyl groups of uronic acid in AGPs and pectins could lead to the formation of the adhesive properties of the mucilage (Huang et al., 2016). Such interactions might be essential in maintaining the structural properties of the RET but also in regulating adhesion and trapping of soilborne microorganisms. AGPs are promising candidates to be involved in early signaling and immune responses within the RET based several indications including: *i*) soluble AGPs could be released by cleavage of GPI-anchored moiety, *ii*) AGPs are involved in the Ca^2+^ signaling pathways, *iii*) enzymatic degradation by microorganisms releasing damage associated molecular pattern (DAMP) and *iv*) acting as of extracellular cargoe receptors initiating endocytosis (Wang et al., 2019). Therefore, to assess the precise contribution of AGPs in pea root protection the role of individual AGPs should be elucidated using transgenic lines affected in the protein backbone and/or in the glycan structure.

## 4 Future prospects for pea protection against root rot disease

To date, there is no registered chemical substances directed against *A. euteiches* and their use is not part of a sustainable agriculture. Furthermore, it should be taken into consideration that fungicides can also affect mycorrhizal fungal establishment leading to reductions in pea nitrogen fixation (Chang et al., 2013). Despites increasing progress in breeding for root rot disease resistance, no complete resistant pea cultivars are available (Pilet-Nayel et al., 2005; Lavaud et al., 2015; Lavaud et al., 2016). Avoidance of infested fields remains the more reliable method to manage root rot disease and assays were designed in order to evaluate the level of soil infectivity before subsequent pea sawing (Sauvage et al., 2007; Moussart et al., 2009; Gangneux et al., 2014). Oospores, the primary source of inoculum, can survive several years in soils before infesting host species such as pea (Papavizas and Ayers, 1974). Consequently, long-term rotations are necessary to avoid pea crop infestation. It is now recognized that several pathogens including *A. euteiches*, *Fusarium *spp., *Phytophthora* spp.*, Pythium* spp., or *Rhizoctonia* spp. interact synergistically to infect the plant forming the pea root rot complex (PRRC) that aggravates pea root rot disease. The involvement of multi-species pathogens in the PRRC is a major limiting factor for plant breeding making complete pea resistance highly complex (Chatterton et al., 2019; Wille et al., 2021; Wu et al., 2022). However, plant beneficial microorganisms such as arbuscular mycorrhizal fungi (AMF) *Glomus intraradices* and *Glomus claroideum* were reported to slightly increase pea tolerance to root rot development (Thygesen et al., 2004). Field experiments suggest that AMF influence the reproductive stage of *A. euteiches* thus limiting the production of oospores within the infected plant tissues and their subsequent release into the soil (Bødker et al., 2002). Stimulating the immune defenses of pea was reported to be an interesting lever against root rot disease. Elicitation with oligogalacturonide fractions shown a protective effect in pea, with an induction of plant defense leading to a reduction in infection (Selim et al., 2017). The difficulties in controlling root rot disease have prompted a search for biological alternatives including the possibility of inter-cropping. French faba bean (*Vicia faba* L.) is a legume species recognized to be tolerant to root rot disease. Recently, root exudates from faba bean were shown to have a repellent effect on zoospores of *A. euteiches* (Laloum et al., 2021). Interestingly, experiments involving pea and faba bean co-cultivation resulted in reduced infection of root pea by *A. euteiches*. Similar data were also obtained when pea seedlings were inoculated with *A. euteiches* and cultivated in the presence of faba bean exudates. These findings highlight the *in vitro* protective effect of faba bean against pea root rot disease at early stages of infection. It is therefore of importance to investigate such protection under field conditions but also at a latest stage of infection to assess potential allopathic effects of faba bean. This study offers promising applications for the development of novel biocontrol agents and/or inter-cropping strategies for pea crop management. Extracts or root exudates from faba bean could be used in agriculture as bioactive natural compounds to improve pea protection against root rot disease caused by *A. euteiches* and the associated PRRC. It is also important, in order to contribute to sustainable agriculture, to investigate belowground interactions between pea roots and allopathic plant species with a special focus on the involvement of root AC-DCs and AGPs.

## Author contributions

MF conceived and designed the figures. MV wrote the first draft. M-LF-G, AD, MF, VL, BP, AG and MV edited and improved the manuscript. All authors contributed to the article and approved the submitted version.

## References

[B1] Bødker L. Kjøller R. Kristensen K. Rosendahl S. (2002). Interactions between indigenous arbuscular mycorrhizal fungi and *Aphanomyces euteiches* in field-grown pea. Mycorrhiza 12, 7–12. doi: 10.1007/s00572-001-0139-4 11968948

[B2] Balmer D. de Papajewski D. V. Planchamp C. Glauser G. Mauch-Mani B. (2013). Induced resistance in maize is based on organ-specific defence responses. Plant J. 74, 213–225. doi: 10.1111/tpj.12114 23302050

[B3] Bozsoki Z. Cheng J. Feng F. Gysel K. Vinther M. Andersen K. R.. (2017). Receptor-mediated chitin perception in legume roots is functionally separable from nod factor perception. Proc. Natl. Acad. Sci. 114, E8118–E8127. doi: 10.1073/pnas.1706795114 28874587 PMC5617283

[B4] Brigham L. A. Woo H.-H. Nicoll M. Hawes M. C. (1995). Differential expression of proteins and mRNAs from border cells and root tips of pea. Plant Physiol. 109, 457–463. doi: 10.1104/pp.109.2.457 12228604 PMC157607

[B5] Burger T. G. Zhang Y. (2019). Recent progress in the utilization of pea protein as an emulsifier for food applications. Trends Food Sci. Technol. 86, 25–33. doi: 10.1016/j.tifs.2019.02.007

[B6] Cannesan M.-A. Durand C. Burel C. Gangneux C. Lerouge P. Ishii T.. (2012). Effect of arabinogalactan proteins from the root caps of pea and *Brassica napus* on *Aphanomyces euteiches* zoospore chemotaxis and germination. Plant Physiol. 159, 1658–1670. doi: 10.1104/pp.112.198507 22645070 PMC3425204

[B7] Cannesan M.-A. Gangneux C. Lanoue A. Giron D. Laval K. Hawes M. C.. (2011). Association between border cell responses and localized root infection by pathogenic *Aphanomyces euteiches* . Ann. Bot. 108, 459–469. doi: 10.1093/aob/mcr177 21807690 PMC3158693

[B8] Chaboud A. (1983). Isolation, purification and chemical composition of maize root cap slime. Plant Soil 73, 395–402. doi: 10.1007/BF02184316

[B9] Chang K. F. Hwang S. F. Ahmed H. U. Gossen B. D. Turnbull G. D. Strelkov S. E. (2013). Management strategies to reduce losses caused by fusarium seedling blight of field pea. Can. J. Plant Sci. 93, 619–625. doi: 10.4141/cjps2012-293

[B10] Chatterton S. Harding M. W. Bowness R. Mclaren D. L. Banniza S. Gossen B. D. (2019). Importance and causal agents of root rot on field pea and lentil on the Canadian prairies 2014–2017. Can. J. Plant Pathol. 41, 98–114. doi: 10.1080/07060661.2018.1547792

[B11] Chuberre C. Plancot B. Driouich A. Moore J. P. Bardor M. Gugi B.. (2018). Plant immunity is compartmentalized and specialized in roots. Front. Plant Sci. 9. doi: 10.3389/fpls.2018.01692 PMC6279857 30546372

[B12] Day L. (2013). Proteins from land plants – potential resources for human nutrition and food security. Trends Food Sci. Technol. 32, 25–42. doi: 10.1016/j.tifs.2013.05.005

[B13] Driouich A. Follet-Gueye M.-L. Vicré M. Hawes M. C. (2013). Root border cells and secretions as critical elements in plant host defense. Curr. Opin. Plant Biol. 16, 489–495. doi: 10.1016/j.pbi.2013.06.010 23856080

[B14] Driouich A. Smith C. Ropitaux M. Chambard M. Boulogne I. Bernard S.. (2019). Root extracellular traps versus neutrophil extracellular traps in host defence, a case of functional convergence? Biol. Rev 94, 1685–1700. doi: 10.1111/brv.12522 31134732

[B15] Durand C. Vicré-Gibouin M. Follet-Gueye M.-L. Duponchel L. Moreau M. Lerouge P.. (2009). The organization pattern of root border-like cells of arabidopsis is dependent on cell wall homogalacturonan. Plant Physiol. 150, 1411–1421. doi: 10.1104/pp.109.136382 19448034 PMC2705035

[B16] Erb M. Balmer D. De Lange E. S. Von Merey G. Planchamp C. Robert C.. (2011). Synergies and trade-offs between insect and pathogen resistance in maize leaves and roots. Plant Cell Environ. 34, 1088–1103. doi: 10.1111/j.1365-3040.2011.02307.x 21410707

[B17] FAO (2018) FAOSTAT online database. Available at: https://www.fao.org/faostat.

[B18] Foyer C. H. Lam H.-M. Nguyen H. T. Siddique K. H. M. Varshney R. K. Colmer T. D.. (2016). Neglecting legumes has compromised human health and sustainable food production. Nat. Plants 2, 1–10. doi: 10.1038/nplants.2016.112 28221372

[B19] Gangneux C. Cannesan M.-A. Bressan M. Castel L. Moussart A. Vicré-Gibouin M.. (2014). A sensitive assay for rapid detection and quantification of aphanomyces euteiches in soil. Am. Phytopathological Soc. 104, 1138–1147. doi: 10.1094/PHYTO-09-13-0265-R 24835221

[B20] Gaspar Y. M. Nam J. Schultz C. J. Lee L.-Y. Gilson P. R. Gelvin S. B.. (2004). Characterization of the arabidopsis lysine-rich arabinogalactan-protein AtAGP17 mutant (rat1) that results in a decreased efficiency of agrobacterium transformation. Plant Physiol. 135, 2162–2171. doi: 10.1104/pp.104.045542 15286287 PMC520787

[B21] Gaulin E. Jacquet C. Bottin A. Dumas B. (2007). Root rot disease of legumes caused by *Aphanomyces euteiches* . Mol. Plant Pathol. 8, 539–548. doi: 10.1111/j.1364-3703.2007.00413.x 20507520

[B22] Gibert S. (2021). Root rots in pea, characterisation and biocontrol of the parasitic complex of telluric origin including *Aphanomyces euteiches* . Institut Natl. la Recherche Agronomique.

[B23] Gunawardena U. Hawes M. C. (2002). Tissue specific localization of root infection by fungal pathogens: Role of root border cells. MPMI 15, 1128–1136. doi: 10.1094/MPMI.2002.15.11.1128 12423018

[B24] Gunawardena U. Rodriguez M. Straney D. Romeo J. T. VanEtten H. D. Hawes M. C. (2005). Tissue-specific localization of pea root infection by *Nectria haematococca.* mechanisms and consequences. Plant Physiol. 137, 1363–1374. doi: 10.1104/pp.104.056366 15778461 PMC1088327

[B25] Hawes M. C. (1990). Living plant cells released from the root cap: A regulator of microbial populations in the rhizosphere? Plant Soil 129, 19–27. doi: 10.1007/BF00011687

[B26] Hawes M. C. Brigham L. A. (1992). Impact of root border cells on microbial populations in the rhizosphere. Adv. Plant Pathol. 8, 119–148.

[B27] Hawes M. C. Brigham L. A. Wen F. Woo H. H. Zhu Y. (1998). Function of root border cells in plant health: pioneers in the rhizosphere. Annu. Rev. Phytopathol. 36, 311–327. doi: 10.1146/annurev.phyto.36.1.311 15012503

[B28] Hawes M. C. Gunawardena U. Miyasaka S. Zhao X. (2000). The role of root border cells in plant defense. Trends Plant Sci. 5, 128–133. doi: 10.1016/S1360-1385(00)01556-9 10707079

[B29] Hawes M. C. Lin H.-J. (1990). Correlation of pectolytic enzyme activity with the programmed release of cells from root caps of pea (*Pisum sativum*) 1. Plant Physiol. 94, 1855–1859. doi: 10.1104/pp.94.4.1855 16667927 PMC1077464

[B30] Hawes M. C. Pueppke S. G. (1986). Sloughed peripheral root cap cells: Yield from different species and callus formation from single cells. Am. J. Bot. 73, 1466–1473. doi: 10.1002/j.1537-2197.1986.tb10892.x

[B31] Hiltpold I. Jaffuel G. Turlings T. C. J. (2015). The dual effects of root-cap exudates on nematodes: from quiescence in plant-parasitic nematodes to frenzy in entomopathogenic nematodes. J. Exp. Bot. 66, 603–611. doi: 10.1093/jxb/eru345 25165149 PMC4286403

[B32] Huang Y. Wang Y. Tan L. Sun L. Petrosino J. Cui M.-Z.. (2016). Nanospherical arabinogalactan proteins are a key component of the high-strength adhesive secreted by English ivy. Proc. Natl. Acad. Sci. 113, E3193–E3202. doi: 10.1073/pnas.1600406113 27217558 PMC4988582

[B33] Immerzeel P. Eppink M. M. De Vries S. C. Schols H. A. Voragen A. G. J. (2006). Carrot arabinogalactan proteins are interlinked with pectins. Physiologia Plantarum 128, 18–28. doi: 10.1111/j.1399-3054.2006.00712.x

[B34] Jensen J. K. Sørensen S. O. Harholt J. Geshi N. Sakuragi Y. Møller I.. (2008). Identification of a xylogalacturonan xylosyltransferase involved in pectin biosynthesis in arabidopsis. Plant Cell 20, 1289–1302. doi: 10.1105/tpc.107.050906 18460606 PMC2438468

[B35] Karaca A. C. Low N. Nickerson M. (2011). Emulsifying properties of chickpea, faba bean, lentil and pea proteins produced by isoelectric precipitation and salt extraction. Food Res. Int. 44, 2742–2750. doi: 10.1016/j.foodres.2011.06.012

[B36] Knee E. M. Gong F. C. Gao M. Teplitski M. Jones A. R. Foxworthy A. (2001). Root mucilage from pea and its utilization by rhizosphere bacteria as a sole carbon source. Mol. Plant Microbe Interact. 14, 775–784. doi: 10.1094/MPMI.2001.14.6.775 11386373

[B37] Krefting J. (2017). The appeal of pea protein. J. Renal Nutr. 27, e31–e33. doi: 10.1053/j.jrn.2017.06.009

[B38] Laloum Y. Gangneux C. Gügi B. Lanoue A. Munsch T. Blum A.. (2021). Faba bean root exudates alter pea root colonization by the oomycete *Aphanomyces euteiches* at early stages of infection. Plant Sci. 312, 111032. doi: 10.1016/j.plantsci.2021.111032 34620436

[B39] Lavaud C. Baviere M. Le Roy G. Hervé M. R. Moussart A. Delourme R.. (2016). Single and multiple resistance QTL delay symptom appearance and slow down root colonization by *Aphanomyces euteiches* in pea near isogenic lines. BMC Plant Biol. 16, 166. doi: 10.1186/s12870-016-0822-4 27465043 PMC4964060

[B40] Lavaud C. Lesné A. Piriou C. Le Roy G. Boutet G. Moussart A.. (2015). Validation of QTL for resistance to *Aphanomyces euteiches* in different pea genetic backgrounds using near-isogenic lines. Theor. Appl. Genet. 128, 2273–2288. doi: 10.1007/s00122-015-2583-0 26215183

[B41] Moussart A. Wicker E. Le Delliou B. Abelard J.-M. Esnault R. Lemarchand E.. (2009). Spatial distribution of *Aphanomyces euteiches* inoculum in a naturally infested pea field. Eur. J. Plant Pathol. 123, 153–158. doi: 10.1007/s10658-008-9350-x

[B42] Mravec J. Kračun S. K. Rydahl M. G. Westereng B. Pontiggia D. De Lorenzo G.. (2017). An oligogalacturonide-derived molecular probe demonstrates the dynamics of calcium-mediated pectin complexation in cell walls of tip-growing structures. Plant J. 91, 534–546. doi: 10.1111/tpj.13574 28419587

[B43] Nazir N. Badri Z. A. Bhat N. A. Bhat F. A. Sultan P. Bhat T. A.. (2022). Effect of the combination of biological, chemical control and agronomic technique in integrated management pea root rot and its productivity. Sci. Rep. 12, 11348. doi: 10.1038/s41598-022-15580-1 35790796 PMC9256638

[B44] Nguema-Ona E. Vicré-Gibouin M. Cannesan M.-A. Driouich A. (2013). Arabinogalactan proteins in root–microbe interactions. Trends Plant Sci. 18, 440–449. doi: 10.1016/j.tplants.2013.03.006 23623239

[B45] Oosterveld A. Voragen A. G. J. Schols H. A. (2002). Characterization of hop pectins shows the presence of an arabinogalactan-protein. Carbohydr. Polymers 49, 407–413. doi: 10.1016/S0144-8617(01)00350-2

[B46] Papavizas G. C. Ayers W. A. (1974). Aphanomyces species and their root diseases in pea and sugarbeet - a review, technical bulletin, agricultural research service. United States Department of Agriculture

[B47] Pilet-Nayel M. L. Muehlbauer F. J. McGee R. J. Kraft J. M. Baranger A. Coyne C. J. (2005). Consistent quantitative trait loci in pea for partial resistance to *Aphanomyces euteiches* isolates from the united states and France. Phytopathology® 95, 1287–1293. doi: 10.1094/PHYTO-95-1287 18943359

[B48] Rougier M. Chaboud A. (1985). Mucilages secreted by roots and their biological function. Israel J. Bot. 34, 129–146. doi: 10.1080/0021213X.1985.10677017

[B49] Sauvage H. Moussart A. Bois F. Tivoli B. Barray S. Laval K. (2007). Development of a molecular method to detect and quantify aphanomyces euteiches in soil. FEMS Microbiol. Lett. 273, 64–69. doi: 10.1111/j.1574-6968.2007.00784.x 17559389

[B50] Selim S. Sanssené J. Rossard S. Courtois J. (2017). Systemic induction of the defensin and phytoalexin pisatin pathways in pea (*Pisum sativum*) against *Aphanomyces euteiches* by acetylated and nonacetylated oligogalacturonides. Molecules 22, 1017. doi: 10.3390/molecules22061017 28629201 PMC6152630

[B51] Sherwood R. T. (1987). Papilla formation in corn root-cap cells and leaves inoculated with *Colletotricum graminicola* . Phytopathology 77, 930–934.

[B52] Sun X. D. Arntfield S. D. (2012). Gelation properties of myofibrillar/pea protein mixtures induced by transglutaminase crosslinking. Food Hydrocolloids 27, 394–400. doi: 10.1016/j.foodhyd.2011.11.001

[B53] Thygesen K. Larsen J. Bødker L. (2004). Arbuscular mycorrhizal fungi reduce development of pea root-rot caused by *Aphanomyces euteiches* using oospores as pathogen inoculum. Eur. J. Plant Pathol. 110, 411–419. doi: 10.1023/B:EJPP.0000021070.61574.8b

[B54] Vicré M. Santaella C. Blanchet S. Gateau A. Driouich A. (2005). Root border-like cells of arabidopsis. microscopical characterization and role in the interaction with rhizobacteria. Plant Physiol. 138, 998–1008. doi: 10.1104/pp.104.051813 15908608 PMC1150414

[B55] Wang L. Cheng M. Yang Q. Li J. Wang X. Zhou Q.. (2019). Arabinogalactan protein–rare earth element complexes activate plant endocytosis. Proc. Natl. Acad. Sci. 116, 14349–14357. doi: 10.1073/pnas.1902532116 31239335 PMC6628639

[B56] Wen F. VanEtten H. D. Tsaprailis G. Hawes M. C. (2007). Extracellular proteins in pea root tip and border cell exudates. Plant Physiol. 143, 773–783. doi: 10.1104/pp.106.091637 17142479 PMC1803736

[B57] Wen F. White G. J. VanEtten H. D. Xiong Z. Hawes M. C. (2009). Extracellular DNA is required for root tip resistance to fungal infection. Plant Physiol. 151, 820–829. doi: 10.1104/pp.109.142067 19700564 PMC2754639

[B58] Wen F. Zhu Y. Hawes M. C. (1999). Effect of pectin methylesterase gene expression on pea root development. Plant Cell 11, 1129–1140. doi: 10.1105/tpc.11.6.1129 10368183 PMC144245

[B59] Wille L. Kurmann M. Messmer M. M. Studer B. Hohmann P. (2021). Untangling the pea root rot complex reveals microbial markers for plant health. Front. Plant Sci. 12. doi: 10.3389/fpls.2021.737820 PMC8545811 34712258

[B60] Wu L. Fredua-Agyeman R. Strelkov S. E. Chang K.-F. Hwang S.-F. (2022). Identification of novel genes associated with partial resistance to aphanomyces root rot in field pea by BSR-seq analysis. Int. J. Mol. Sci. 23, 9744. doi: 10.3390/ijms23179744 36077139 PMC9456226

[B61] Xie F. Williams A. Edwards A. Downie J. A. (2012). A plant arabinogalactan-like glycoprotein promotes a novel type of polar surface attachment by *Rhizobium leguminosarum* . MPMI 25, 250–258. doi: 10.1094/MPMI-08-11-0211 21995765

[B62] Zhao X. Schmitt M. Hawes M. C. (2000). Species-dependent effects of border cell and root tip exudates on nematode behavior. Phytopathology® 90, 1239–1245. doi: 10.1094/PHYTO.2000.90.11.1239 18944426

[B63] Zhu Y. Pierson L. S. III Hawes M. C. (1997). Induction of microbial genes for pathogenesis and symbiosis by chemicals from root border cells. Plant Physiol. 115, 1691–1698. doi: 10.1104/pp.115.4.1691 9414568 PMC158635

